# Pharmacy of the Greeks and Romans

**Published:** 1851-05

**Authors:** 


Art. II.—*Pharmacy of the Greeks and Romans.* Translated from the French of M. Cap, by W. H. Cobb, M. D.

Among the Greeks, Hippocrates sums up pretty completely the state of pharmacy; while among the Romans, Galen furnishes us with the most exact account. In the writings of these two masters of their science, then, must we look for the principal features of the history of our profession, at the epochs when it was in the most advanced state, in ancient times. Many catalogues of the simple medicines used by Hippocrates have been published. Virey, and after him Daniel Leclerc, collected a catalogue numbering about three hundred substances. K. Sprengel has given a much more exact account of Hippocrates. Dr. James, in the introduction to his Medical Dictionary, furnishes us with a long list of medicines, simple or compound, made use of by Hippocrates—a list drawn equally from the writings of Polybius, Thessalus, Dracon, and others who have, as we know, added their works to those of their master: and Fourcroy has extracted from them some general remarks, interesting both as regards the history of our science and its application.

Among the medicines comprised in these different lists, we notice quite a large number without any well-marked action upon the human economy. Leeches do not figure there, and many drugs known at the time of Galen occupy no place there.

They used internally certain preparations of copper; they were acquainted with canella, cardamon and pepper; from Egypt they received opium and scammony; butter was scarce, and but little known; cantharides and other insects were used internally; they were acquainted with the exhibition of the fetid gums, in the treatment of the neuroses and hysteria; they seasoned their food with the umbelliferous and labiate plants. Into the composition of their collyria, entered the bile of many animals; but by the term collyrium, they designated, as we shall see hereafter, a very different class of remedies from those which we signify at the present day by the same name.

With Hippocrates, therapeutics was confounded to a certain extent with dietetics, for he expressly says "that the food and drinks which man uses in a state of health, ought also to serve him when he is sick." He examines, in fact, the greater part of aliments in this double point of view, and his account of the qualities of the flesh of the dog, fox, horse and ass, would lead us to think that these viands were then in general use.

The broth of barley, diet, and repose, were the means which he employed at the onset of acute diseases. He added to the ptisan of barley a little vinegar, oil and salt, and occasionally anise and leek. The ordinary beverage of the sick was composed of 8 parts of water to 1 of honey, to which was sometimes added vinegar. The cyceon was a drink, into which entered rice, the seeds of the anise, celery, coriander, wine and wheaten flour.

Hippocrates employed milk and whey, both as food and as medicine; not only the milk of the cow, but of the goat, mare and she-ass, which he prescribed in a large dose as a laxative.

Sudorific medicines were then unknown. To excite perspiration, they employed baths, fumigations, frictions, and the heat of the sweating room. The diuretics used internally were sweet wine, garlic, onion, leek, cucumber, melon, citron, celery, bas trefoil, fennel, capillaire solanum, oxymel, hydromel, and the cantharides deprived of their legs and wings, and mixed with honey and wine. Narcotics, or rather the somniferæ of Hippocrates, were the poppy, opium, the euphorbia peplus, mandragora and henbane; his febrifuges, absinthium, little centaury, and some other indigenous bitters. To excite emesis, he employed the assarabacca, the white hellebore, and a plant named sesamoides, and which Dioscorides calls the Hellebore of Anticyrus; or he gave them to drink a large quantity of some laxative, followed by a decoction of lentils and hyssop with honey and vinegar.

His laxatives were mercury, cabbage in decoction, whey, and the milk of the she-ass, salted. He employed suppositories and lavements. The suppositories were composed of honey, mercury, salt, nitre, the powder of colocynth, and other irritating substances; they were sometimes round, like a ball, and sometimes of an elongated form. The lavements were composed of a decoction of the leaves of the beet, with the addition of honey, nitre, and other laxative substances.

The purgatives of Hippocrates were numerous. He employed black and white hellebore, the berries of the daphne mezereum, the daphne thymelæa, euphorbia peplus, thapsia, swallow thorn, elaterium, colocynth, scammony, magnesian stone (a variety of loadstone), cnicus, etc.

He admitted purgatives, directed specifically to the bile, phlegm, melancholy and dropsy. To clear the head from its affections, he caused his patients to inspire by the nostrils, the juice of the celery, divers aromatics, and powders composed of myrrh, the flour of brass (oxide of copper), and white hellebore; as an expectorant, he applied to the base of the tongue the root of the arum triphyllum, boiled in water, with honey, oil and salt.

*External Medicaments.*—Fomentations were arranged under two heads, wet and dry. The first were partial or local baths of lukewarm water, or of the decoctions of certain plants. They also applied upon the part affected a leathern bottle, bladder, or some kind of vessel, filled with warm water; at other times a sponge, soaked in a decoction of barley, bran, or the seeds of the heath peas. The dry fomentations were made of salt, roasted millet, or of aromatic substances, enclosed in little bags and applied upon the part affected. For fumigations, they employed the vapor of water, either pure or impregnated with divers medicinal substances; for example, they heated pieces of iron, plunged them into urine, and directed the vapor upon the part. They were also made with the smoke of resins, bitumens, and aromatics, burned.

The gargles recommended by Hippocrates in diseases of the mouth and throat, were composed of a decoction of origan, savory, celery, mint with a little nitre, honey and vinegar. The oils and unguents were employed to anoint the body, soften tumors, and dress wounds. The oils were pure or compound: the pure oil was that of the olive; the compound oils, as those of roses and myrtle, were obtained by infusion. The susinum was prepared from the white lily and aromatics; the narcissum had for its base the narcissus. The netopum and the metopium, cited by Dioscorides, and the mendosium, of which Galen speaks, were preparations of the same class, even more complicated. Hippocrates bestowed the name of cerate upon an unguent composed of oil and wax; another species of cerate was prepared of the fat of the goose, turpentine, the resin of the mastich, wax, and the oil of roses. When to the simple cerate pitch was added, to give it consistence, it obtained the name of ceropissus.

The cataplasms were composed of vegetable powders, mingled with the juice of plants, to which was sometimes added a small quantity of oil or of an unguent. In quinsy, they applied to the neck a cataplasm of the meal of barley with wine and oil. The emollient cataplasms were composed of the leaves of the beet, cooked in water, with the addition of the leaves of the olive, fig, or oak.

*Internal Medicines.*—The fluid preparations administered internally, included decoctions, infusions of vegetable substances, in which they sometimes dissolved powders, the juice of plants, the mixtures of wine, honey, oil, vinegar, and other simple and compound liquids. They also employed the medicinal wines prepared by infusion.

The solid preparations were composed of the inspissated juices (extracts), gums, resin, and various powders, the whole mixed with honey and other ingredients, and fashioned into the proper shape. This is, then, we see, the point of commencement of antidotes and electuaries, preparations which in subsequent ages, and even in our own times, occupied so prominent a place in pharmacy. The collyria were solid masses, of the length and shape of the finger, intended to be introduced into a cavity, like suppositories and pessaries. The troches were of the same shape, and used for the same purpose, as at the present day. The pectoral remedies were medicines of a soft consistence, placed upon the tongue and swallowed slowly; honey and various aromatic or mucilaginous powders formed their base. Finally, they were acquainted with the mels, oxymels, and confections, but not with the syrups, whose name, derived from the Arabic language, denotes a much more recent origin. In scanning the writings of this epoch, we find no mention of the different operations employed for obiaining all these products. That the manipulations could not have been very complicated, one can very readily perceive. Digestion, infusion, decoction, the expression of the juices and their inspissation by heat, and pulverization, were the methods most frequently employed. Evaporation, calcination, fusion, perhaps even sublimation, were known to the operators of that period. But if we except lixiviation, the crystallization of salts, and the extraction of metals, they practised no one operation truly chemical, nor did they have any idea of combinations of this nature. As the healing art had not as yet been divided into its different branches, the physician prepared the medicines, as well as prescribed them. Hippocrates prepared with his own hands the greater number of his medicinal agents, or had them prepared by his servants, who were instructed to that effect; so also did all the other physicians of his time. We know well that he carried his medicines with him when called by the inhabitants of Abdera to cure Democritus.

As we leave farther and farther behind the epoch in which the school of Cos shone forth, and approach that in which the schools of dogmas and methods reigned, we cannot fail to mark the increasing tendency to an indefinite complication, which has found its way into therapeutics and pharmacy.

The summum bonum of the art, for a physician of that period to attain, and sometimes the chief element of his reputation, was the composition of an electuary, or of an antidote, in which was heaped together a crowd of substances endowed with dissimilar properties, bestowing upon it remedial virtues in a great number of diseases.

The physicians of that period believed that each medicinal substance possessed, relatively to a given disease, an absolute curative property, but that this property was accompanied, with regard to the different organs, by a physical action, often in direct opposition to its medical efficacy. Consequently, they provided each principal drug, with an escort of many others, intended, some to increase its medical activity, and others to modify its influence upon the organism; and his chief care was expended upon the choice of ingredients which should serve as a bond of union, a vehicle for all this mass, for this strange chaos of which the electuary was composed.

We have often said that the word electuary was derived from the Latin verb *eligere*, to choose; nevertheless, Cœlius Aurelianus, who flourished about the time of Galen, places electuaries by the side of the eclegmes, whose etymology we have given above, as derived from the Greeks, and gave to them the title of electarium, whence most probably arose electuarium.

As for the word antidote, (anti dolos, given against,) it is a generic term, applicable to all compound medicines. Galen called all medicines administered internally, antidotes; thence, the word antidotary was for a long time employed synonymously with dispensary, or pharmacopœia. Later, this term signified, as it does at the present time, a remedy given to counteract a poison. At this epoch, there were antidotes against phthisis, contusions, colic, calculi, pleurisy, gout, etc. Galen divided them into three classes: remedies against poisons, against the bite of venomous animals, and against excesses of all kinds. To his eyes, the Theriaca Andromachi united all these three virtues. The consistence of the electuaries and the solid antidotes was about the same; their taste often disagreeable. From them they made little balls of different sizes; those that had the form of a berry, or a pea, were named globuli—glomerami et pilulæ (in Greek, kokkos); others, of the shape of a bean, or of the seed of the lupine, were termed pastilli and trochisci, from the Greek word trochiskoi.

The names given by the Greeks and Romans to their medicines, related oftentimes to their uses; thus they called those remedies that were employed against diseases of the lungs, and trachea arteriacæ; those employed for alleviating cough, bechicæ. Pills, placed under the tongue and allowed to melt slowly in the mouth, were styled hippoglottides. The eclegmes of a softer consistence, were composed of gum tragacanth or arabic, of the juice or powder of liquorice, myrrh, honey, wine, saffron, and they sometimes added diacodion or opium. Then they were named anodyne, or paregorica.

The liquid medicines did not differ remarkably from those of Hippocrates. The drinks most employed were infusions, decoctions, and the juices of plants, more or less diluted. Sometimes they disguised the antidote when taken, in water, wine, or hydromel. The liquid medicines generally bore the name of potions. Those obtained by decoction, were the decocta and apozemata. Galen also gave the name of decocta to water which had been boiled and afterwards been cooled in the snow. There were also drinks which they took in the state of health as well as in sickness: thus they drank wine in which were infused wormwood, pepper, and casamum. To these they added honey and other ingredients, which gave the names to these drinks. They also used water in which were boiled apples and roses, with the addition of verjuice, the juice of the pomegranate, the berries of the myrtle, and honey. The drinks compounded of honey, wine, and aromatics, were termed propomata. Paulus Ægineta and Nicolaus Myrepsus have preserved many recipes of them. A variety of propomata, drunk iced, was called recentatum. At the commencement of their repasts they drank compound wines, called condita, to excite the appetite.

Vinum mulsum, or simply mulsum, was a mixture of 4 parts of wine to 1 of honey. The hydromel, composed of water and honey, was called indifferently aqua mulsa, or mulsa. The hydromelon was formed of hydromel and the juice of quinces; to the hydrorosatum they added roses instead of the juice of quinces. When they mixed these four substances, they obtained the rhodomelon and rhodostacton, which bore a close resemblance to our mel rosae. Modern pharmacy has preserved the oxymel, a compound of honey and vinegar, and the oxycrate, a compound of vinegar and water. From honey and verjuice were prepared the omphacomeli; from honey and the juice of the myrtle, the myrtites; and from honey and the juice of the pomegranate, the rhoites. They made similar preparations from the most of fruits. The apomeli was water in which was boiled honey-comb.

Among the remedies applied externally, the oils held the first rank. They were prepared by infusing simples in the oil of the olive, walnut, almond, and sesame; and when the oil was highly charged with the active principles of the plant, they gave it the name of unguentum. This word was applied to every preparation which served to anoint the body, but more especially to the aromatic oils and liquid perfumes. The unguent of roses was the most employed. The unguents were also called acopa, (from a, privative, and kopos, fatigue,) because they were often employed to lighten fatigue. The same appellation was bestowed upon all the preparations used externally for the same purpose, as the mixtures of wax, of honey, of turpentine, of various resins and axunge: of this class was the cereleon, a semi-fluid compound of wax and oil; when aromatics entered into its composition, it was termed myracopa. The cerate contained not only a large proportion of oil, but there were also added divers powders. According to Galen, the cereleon and the acopa were unguents remarkably fluid; next came the cerates, and lastly the plasters. Paulus Ægineta says that the illitiones were even more liquid than the acopa. The oxyrhodines were a mixture of vinegar and oil of roses. The term plaster was applied not merely to those preparations into which entered a larger proportion of wax than into the cerates, but also to those to which were added metallic powders, as litharge, ceruse, the oxide of copper, or even the earths, chalk, etc. Those of the softest consistence were named lissaræ, plasters containing fat, or parygræ, moist plasters; while those in which dry or solid substances predominated, were termed alipanda, plasters without grease, or amolyntha, plasters which did not soil the hands. This last condition was in fact the criterion by which they judged of the proper consistence of the plasters.

From the plasters they formed little masses of a circular shape, and of the length of the finger, which they termed magdalidæ and rotundæ, and which correspond to our magdaleons. The malagma was a compound of gums, aromatics, salts, and other substances, with the addition of a little oil, wax, or axunge. They resembled very closely plasters, and were used to soften and discuss tumors. At other times they were merely gums and resins, dissolved in wine or vinegar. The word malagma was also extended to other remedies of the same consistence. The epithems differed from plasters, and the malagma in this respect alone, that they were not applied upon tumors or wounds, but upon the skin, to act sympathetically (upon it) or by absorption, oftentimes upon the stomach, to strengthen it, or upon recent wounds, as a hæmostatic. Besides the ceropissus, composed of pitch and wax, the dropax were plasters which they applied upon the epidermis, and which they tore from it by force, for the purpose either of reddening the skin, of denuding it, or of acting as a depilatory. They sometimes added salt, sulphur, or the powder of the pyrethrum.

Into the cataplasm, ordinarily formed of plants, meal, or the soft part of bread, cooked in water or other liquid, there sometimes entered oil, honey, the meal of the flax-seed and the fenugreek, figs, yeast, and many other substances, some astringent, others emollient. When they wished to irritate the skin, they added to them mustard in powder, and even cantharides, and then styled them sinapisms.

The smegma of the ancients served to cleanse the skin, to allay pruritus, and even as a dentrifice. Into its composition entered the meal of beans, the seed of melons, the horn of the stag, pumice stone, the bone of the cuttle-fish, antimony, molten lead, sulphur, sal ammoniac, nitre, alum; sometimes stavesacre, hellebore, pepper, cardamon, gums, resins, and the juices of plants, all mixed in honey. With it they rubbed the body before entering the bath. Certain aromatic powders, bearing the name of diapasmata, were spread over the body to absorb the perspiration. When they wished the smegma to produce a depilatory effect, it contained orpiment, sandarac, and quick lime, and received the name of psilothron. Among the opulent Romans, the slaves rubbed the bathers with smegma composed of aromatics of the choicest varieties, brought from all the nations of the world, and enclosed in vessels of gold, alabaster, or crystal. These were the amoricinum, magalium, nardum, and a host of others, to whose names were added the most wonderful epithets, as in the case of electuaries.

The collyrium, so termed from its resemblance to a rat's tail, (colourion, or colobe oura, a tail cut off,) was, as among the Greeks, of a solid consistence, circular in form, and gradually tapering from one end to the other. The tents, plastic masses intended to be introduced into fistulæ or the natural cavities, also bore the name of collyrian; finally they gave the same name to certain compounds, which they dried for their better preservation, and pulverized when they wished to use them. Of this number were certain preparations used in diseases of the eye, and it was by an extension of the term that the name collyrium was applied to the remedies employed for this purpose. The dry collyria were composed of metallic powders, as white lead, oxide of zinc, verdigris, brass, and cadmium, besides saffron, myrrh, opium, aloes, the juice of roses, fennel, and celandine. The liquid collyria were composed of honey, balm of Gilead, the gall of the viper or of the partridge, and the juice of the fennel.

Troches, called also by the Greeks phthoides, were little masses of various forms, generally semi-circular, and not weighing more than a drachm. Some were employed externally, and resembled very closely the collyria in their composition, while others were employed internally. Some they placed in the mouth to perfume the breath; others were burned as perfumes. The troches termed hedychroi were composed chiefly of aromatics. The masses into which they were formed received the name of massa hedychroum.

With the exception of gargles, the remedies used as injections were called clysma; those inspired by the nostrils, errhyna.

We have said that, from the time of Hippocrates up to that of Galen, the materia medica was enriched by quite a number of new remedies. It was during this period that Dioscorides and Pliny collected the numerous documents of which natural history, as relating to medicine, was composed. As to the pharmacy of the same epoch, it is to be found complete in the writings of Galen, who resumed those of Celsus, Andromachus, Asclepiades Pharmacion, Archegenes, and all the authors who had preceded him upon the same subject. Besides the articles which they collected from the two organic kingdoms, they also borrowed much from the mineral. Many salts and metallic oxides entered into the composition of their remedies. They took internally various kinds of salt, pulverized gums, the earth of Lemnos (a variety of chalk), the Jewish stone (the spines of the fossil sea urchins, which they found in Palestine and employed as a remedy for strangury), bloodstone, common salt, nitre, sal ammoniac, fossil salts, potassa obtained by burning rushes and reeds and lixiviating the ashes, medicinal salt, the salt of vipers, the formula for which has been handed down by Pliny and Dioscorides, and finally purgative salts, probably the tartrates, whose activity they augmented by the addition of scammony.

The mineral waters already enjoyed pretty general reputation. They were used as baths, and taken internally. Archigenes had classed them, after their principal constituents, into aluminous, sulphurous, bitumenous and saline. The Greeks called them autophue, which corresponds to the Latin aquæ naturales, or sponte nascentes.

The pharmaceutical manipulations were but little more complicated than at the time of Hippocrates. However, Galen makes mention of the water bath, which he terms diplangium, diploma, vas duplex; he was acquainted with sublimation and distillation per descensum. We have stated in a previous part of this sketch that they were also acquainted with the ambix, from which at a later period was derived the word alembic. Certain utensils employed at this time differed but little from those of the present day: they used mortars, stones for pulverizing, sieves, knives, scissors, rasps, spatulæ, presses, basins, and jars of every possible form, for the preservation of the different medicines. The whole advancement of the art at this period seemed to be concentrated upon this point, that of combining in varied proportions, and with a great number of accessory remedies, agents of the most heterogenous characters, for the purpose of overwhelming, by a single blow, diseases of the most opposite nature, and even of combatting beforehand, by preventive means, morbific causes not yet come into existence.

